# Seizure Prophylaxis Following Moderate to Severe Traumatic Brain Injury: Retrospective Investigation of Clinical Practice and the Impact of Clinical Guidelines

**DOI:** 10.7759/cureus.7709

**Published:** 2020-04-17

**Authors:** Heather Nichol, John Boyd, Jessica Trier

**Affiliations:** 1 Physical Medicine and Rehabilitation, Queen's University, Kingston, CAN; 2 Critical Care Medicine, Queen's University, Kingston, CAN

**Keywords:** traumatic brain injury (tbi), seizure, anticonvulsant, seizure prophylaxis

## Abstract

Background

Post-traumatic seizure (PTS) is a major complication of traumatic brain injury (TBI). However, there has been controversy in the literature regarding whether anticonvulsants should be used prophylactically to prevent it, and there is significant variability in practice. The objective of this study is to describe seizure prophylaxis practices after moderate to severe TBI and to determine whether the use of seizure prophylaxis increased following the recommendations of the Quebec Institut National d'Excellence en Santé et Services Sociaux and the Ontario Neurotrauma Foundation (INESSS-ONF) guidelines. This study will also compare the characteristics of patients who receive the recommended prophylaxis and those who do not.

Methods

All adult patients admitted to a level-1 trauma centre for moderate to severe TBI were eligible for this study (n = 96). Medical records including patient age, sex, Glasgow Coma Scale (GCS) score, mechanism of injury, and occurrence of PTS were reviewed in a retrospective manner regarding the administration of recommended seizure prophylaxis.

Results

The proportion of patients receiving the recommended seizure prophylaxis was 8%. There was no significant increase after the release of the INESSS-ONF guidelines (p: 0.38). There were no significant differences in demographics, injury characteristics, or rates of early PTS between patients receiving the recommended prophylaxis and those not receiving it (p: >0.05).

Conclusion

The results indicate that the use of the recommended seizure prophylaxis after moderate to severe TBI is low and that the release of the INESSS-ONF guidelines did not increase its use. Patient and injury factors do not appear to influence the use of seizure prophylaxis. These results highlight variability in seizure prophylaxis practices and the importance of understanding local practice patterns. Implementation strategies should be identified to increase adherence to the recommendations and improve patient care.

## Introduction

Traumatic brain injury (TBI) is a leading cause of death and disability worldwide. An estimated 69 million people suffer from TBIs every year, with the highest incidence in North America and Europe [1]; 19% of these injuries, or 13 million cases annually, are classified as moderate or severe TBI, defined by longer loss of consciousness and post-traumatic amnesia, and more significant impairment in the level of consciousness [1,2]. These types of severe injuries confer a significant burden on patients and the healthcare system as they are associated with a higher risk for significant short- and long-term complications [2].

One important complication of TBI is post-traumatic seizures (PTS). PTS are classified into immediate PTS (occurring within 24 hours of injury), early PTS (occurring within the first week post-injury), and late PTS (occurring after one week post-injury). The reported rate of early PTS has ranged from 2-14% [2-5]. Controversy exists as to whether anticonvulsants should be used to prevent PTS. Proponents of seizure prophylaxis point to the relatively high incidence of early PTS and its potential negative impact on brain healing and long-term functional recovery, including predisposition to recurrent seizures, as key reasons for supporting its use [2,4,6-8]. Though the impact of early PTS may be significant, they remain largely theoretical, contributing to the debate around seizure prophylaxis.

Opponents of seizure prophylaxis cite the negative neurobehavioural side effects of anticonvulsants, including cognitive impairment, somnolence, and mood disturbance. They also point out the anticonvulsants' questionable effectiveness in preventing PTS [6]. While a landmark study by Temkin et al. found that phenytoin significantly reduced the rate of early PTS, later studies have yielded mixed results [3,9,10-12]. However, recent meta-analyses have concluded that anticonvulsants reduce the rate of early PTS, but not late PTS [8,13-15]. Despite these findings, concerns still remain regarding the risk-benefit ratio of seizure prophylaxis as studies into the negative side effects of seizure prophylaxis have been scarce. Two studies investigating phenytoin found it to be associated with worse short-term, but not long-term, functional outcomes [3,16]. The use of levetiracetam in place of phenytoin has acquired popularity as it may have fewer negative side effects [4]. Recent meta-analyses comparing levetiracetam to phenytoin have found it to be equally or more effective at preventing early PTS [15,17-19]. However, its side effect profile has not been found to be superior as meta-analyses comparing adverse outcomes have yielded mixed results [15,17-19].

In recent years, two North American practice guidelines for the treatment of moderate to severe TBI have been published, consolidating new evidence in this field and recommending anticonvulsants to prevent early PTS [4,20]. Specifically, the Quebec Institut National d'Excellence en Santé et Services Sociaux and the Ontario Neurotrauma Foundation (INESSS-ONF) has published guidelines recommending “anticonvulsants, particularly phenytoin and levetiracetam, …to reduce the incidence of PTS in the first seven days post-injury” for moderate to severe TBI (level C evidence) [20]. However, despite increasing evidence for the effectiveness of seizure prophylaxis and support from practice guidelines, substantial variability in practice still exists. A large, multicentre European study found significant variability in indications for and duration of seizure prophylaxis and identified that although nearly all centres reported the use of practice guidelines for the management of TBI, fewer than half had policies that included seizure prophylaxis [21,22]. Surveys in the United Kingdom and Ireland have found that seizure prophylaxis is prescribed by fewer than 60% of responding neurosurgeons, among whom only 38-50% prescribe anticonvulsants for the recommended seven days [14,23]. North American retrospective medical record reviews have found seizure prophylaxis rates of 26-65% [24-26]. With such wide variability in practice, it is important to understand local patterns of anticonvulsant use post-TBI to assess the quality and consistency of patient care and to develop effective quality improvement initiatives. This study aims to objectively describe seizure prophylaxis practices post-TBI at a level-1 trauma centre and to determine whether the release of locally developed and targeted INESSS-ONF guidelines increased the use of the recommended seizure prophylaxis.

## Materials and methods

Design, setting and participants

The study design was approved by the Health Sciences and Affiliated Teaching Hospitals Research Ethics Board (HSREB) of Queen’s University. This study reviewed patient health records from Kingston General Hospital (KGH), a level-1 trauma centre in Kingston, Ontario, Canada, in a retrospective cohort design. In accordance with the Queen’s University HSREB policy, we verified that consent for research had not been withdrawn before reviewing patient health records. Patients were eligible for the study if they were admitted to KGH for TBI. Inclusion criteria were as follows: 1) patients aged 18 years and older, 2) those with a diagnosis of TBI and 3) those with the lowest Glasgow Coma Scale (GCS) score of <13 in the first 24 hours post-injury. TBI was defined as a nondegenerative, acquired brain insult due to an external mechanical force. Eligible patients were identified through the hospital’s electronic discharge records by searching for a diagnosis of TBI (including intracranial injury), subdural hematoma, epidural hematoma, closed head injury, and subarachnoid hemorrhage. Exclusion criteria included: 1) anticonvulsant use prior to admission and 2) spontaneous subarachnoid hemorrhage secondary to an aneurysm or arteriovenous malformation rupture. Anticonvulsants were defined as medications used to prevent or reduce the severity of seizures. This study identified the following anticonvulsants: acetazolamide, carbamazepine, clobazam, clonazepam, diazepam, gabapentin, lacosamide, lamotrigine, levetiracetam, lorazepam, nitrazepam, oxcarbazepine, phenytoin, topiramate, valproic acid, and vigabatrin.

Data collection and timeline

The medical records for eligible patients admitted to KGH from October 1 to August 31 in 2015/2016 and 2016/2017 were reviewed (n = 96). September 2016 was omitted as this corresponded to the release of the INESSS-ONF guidelines. The timeline for review was up to seven days post-admission or discharge, whichever occurred first.

Patient medical records were reviewed by a single investigator (HN) for baseline data, including age and gender, and for event data, including mechanism of injury, GCS score and admission time (either before or after guideline release). Outcome data included the type of anticonvulsant ordered, if any, the duration of anticonvulsant use, method of the medication order, including the use of preprinted order sets, and the occurrence of early PTS.

Outcome measures

The primary outcomes were a description of the anticonvulsant treatment administered and the proportion of patients who received the recommended seven-day post-TBI seizure prophylaxis. The secondary outcomes included a comparison of the proportion of patients receiving the recommended seizure prophylaxis before and after the release of the INESSS-ONF guidelines as well as a comparison of the characteristics of patients receiving the recommended seizure prophylaxis and those not receiving it. Parameters for comparison included age, gender, mechanism of injury, GCS score and occurrence of early PTS.

Analysis

The type and duration of anticonvulsant treatment were described, including the number and proportion of patients receiving each anticonvulsant or no anticonvulsant, as well as the number of days of treatment. Patients were then categorized into two groups: those who received the guideline-recommended anticonvulsant prophylaxis (phenytoin or levetiracetam for seven days) and those who did not. Patients who received anticonvulsants for less than or more than seven days post-injury were considered as not receiving the recommended prophylaxis. Patients who had begun the recommended anticonvulsant regimen but died or those whose goals of care were changed to comfort measures only before completing seven days were considered as following the guideline recommendations. The proportion of patients who received the recommended seizure prophylaxis was compared between the before-guideline and after-guideline cohorts using a one-tailed Fisher exact test. When comparing the group of patients who did and did not receive the recommended seizure prophylaxis, parameters measured categorically, including gender, GCS score (dichotomized into injury severity: moderate = 9-12, severe = ≤8), mechanism of injury [motor vehicle collision (MVC), fall or other] and occurrence of PTS were compared using Fisher’s exact test for dichotomous variables and chi-square tests with Yates correction for variables with three or more levels. When comparing age, a continuous variable, in the group of patients who did and did not receive the recommended seizure prophylaxis, data were tested for normality with Shapiro-Wilk tests and compared using a Mann-Whitney U test. A Bonferroni-Holm correction was used for multiple comparisons. Comparison of the rate of PTS excluded patients having immediate seizures occurring prior to arrival to the hospital or before the start of anticonvulsant therapy. Statistical analysis was performed with IBM SPSS Statistics version 22 (IBM, Armonk, NY) and custom statistics programs (created by Dr. Joseph Rochford, McGill University, Montreal, Canada).

Sample size

The sample size was estimated to detect an absolute increase in the proportion of patients receiving the recommended anticonvulsant prophylaxis of 25%, which the authors believe represents a clinically significant increase. With an alpha of 0.05 and a power of 80%, it was estimated that 46 patients per group would be required.

## Results

**Figure 1:**
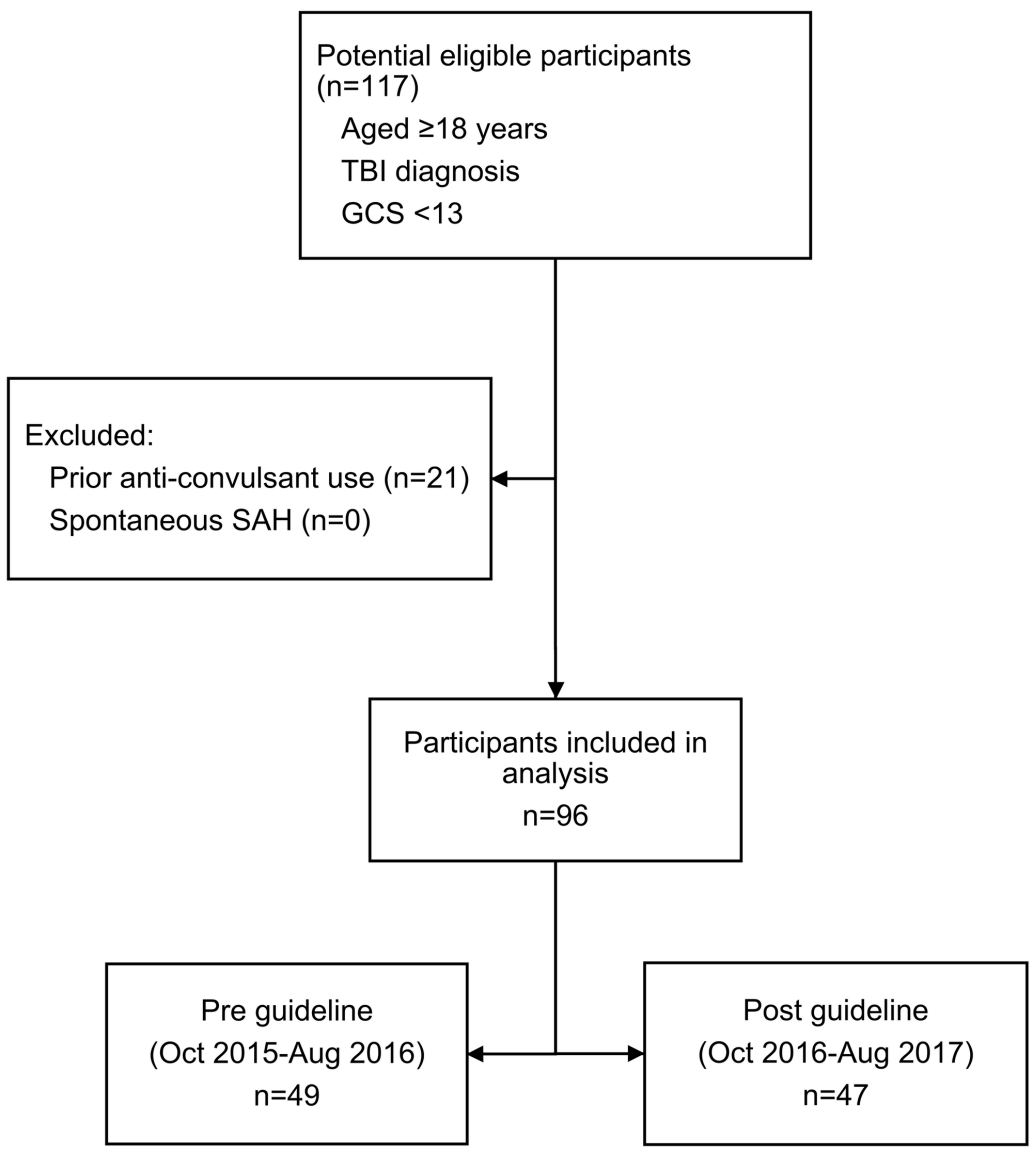
Flow diagram showing retrospective cohort design TBI: traumatic brain injury; GCS: Glasgow Coma Scale; SAH: subarachnoid hemorrhage; Oct: October; Aug: August

A total of 96 eligible patients were identified, consisting of 49 and 47 patients in pre- and post-guideline release groups, respectively (Figure 1). Fourteen patients in each group died within seven days post-injury.

**Figure 2:**
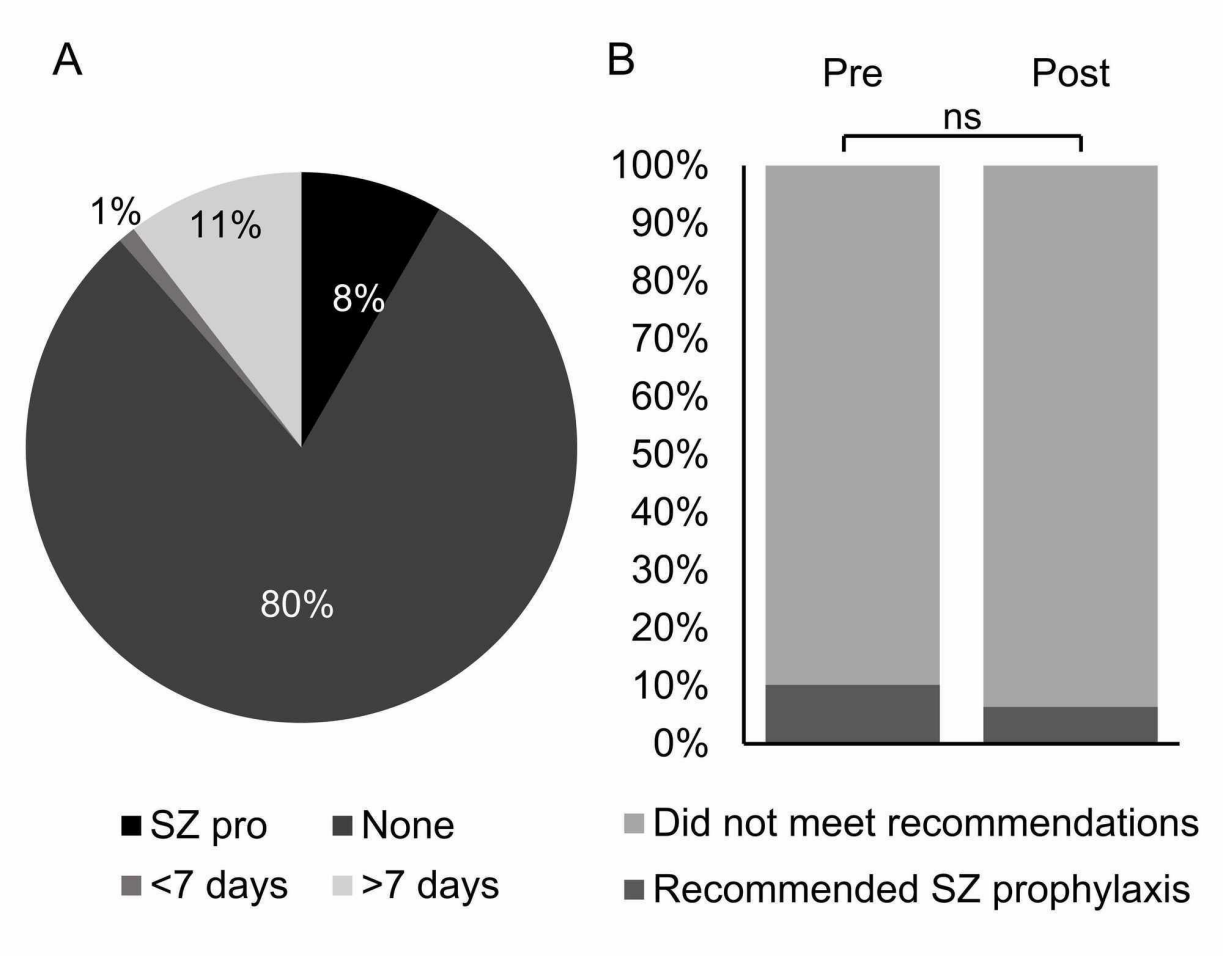
Seizure prophylaxis practices A: proportion of patients receiving the recommended seizure prophylaxis (SZ pro), those not receiving any seizure prophylaxis (None), and those receiving seizure prophylaxis for less than seven days (<7days) or more than seven days (>7 days); B: proportion of patients receiving and not receiving the recommended seizure (SZ) prophylaxis pre- and post-guideline release; ns: not significant (p: >0.05)

Eight of the 96 patients received the recommended seizure prophylaxis (Figure 2A). The majority of these patients received phenytoin, with only one patient receiving levetiracetam. All orders were hand-written on order sheets without the use of preprinted order sets. Of the 88 patients not receiving the recommended seizure prophylaxis, the majority received no anticonvulsant (n = 77), while a minority received an anticonvulsant for more than or less than the recommended seven days (n = 10 and n = 1, respectively) (Figure 2A). When pre- and post-guideline periods were compared, there was no significant increase in the implementation of seizure prophylaxis after the guideline release (p: 0.38, n = 5/49 and n = 3/47 for pre- and post-guideline periods, respectively) (Figure 2B).

A comparison between patients receiving and those not receiving the recommended seizure prophylaxis revealed no significant difference in age, gender, the severity of injury based on GCS score or mechanism of injury (Table 1; p: >0.05 for all). The occurrence of PTS was also recorded. Among those patients receiving the recommended seizure prophylaxis, one experienced an immediate PTS before arriving at the hospital and none experienced an early PTS. Twelve of the 88 patients not receiving the recommended seizure prophylaxis experienced at least one PTS. Six of these patients experienced immediate PTS. The remaining six patients experienced early PTS from day two to four post-injury, including two cases of status epilepticus. Overall, excluding patients who experienced immediate seizures, the rate of early PTS was not significantly different between those receiving and those not receiving the recommended seizure prophylaxis (3% and 7%, respectively; p: >0.05).

**Table 1:** Demographic characteristics and injury parameters of patients receiving and not receiving the recommended seizure prophylaxis *All p-values >0.05 with Bonferroni-Holm correction SEM: standard error of the mean; GCS: Glasgow Coma Scale; MVC: motor vehicle collision

		Seizure prophylaxis	No seizure prophylaxis	P-value*
Age, years, mean ±SEM		48 ±6	55 ±2	0.30
Sex, n (%)				0.46
	Male	4 (50%)	57 (65%)	
	Female	4 (50%)	31 (35%)	
GCS score, n (%)				0.26
	9-12	1 (17%)	31 (35%)	
	≤8	7 (83%)	57 (65%)	
Mechanism of injury, n (%)				0.66
	MVC	2 (25%)	28 (32%)	
	Fall	4 (50%)	53 (60%)	
	Other	2 (25%)	7 (8%)	

## Discussion

Despite the mounting evidence and recently published practice guidelines supporting the use of seizure prophylaxis after moderate to severe TBI, significant variability continues to exist in its practice. The goal of this study was to objectively describe the post-TBI seizure prophylaxis practices at a level-1 trauma centre and to determine whether the release of the locally developed and targeted INESSS-ONF guidelines increased the adherence to the recommended seven-day seizure prophylaxis, as well as to compare the characteristics of those receiving and not receiving the recommended prophylaxis.

Overall, the proportion of patients receiving the recommended seven-day seizure prophylaxis was low at only 8%. Consistent with the recommendations, these patients received phenytoin or levetiracetam [20]. The majority of patients received insufficient seizure prophylaxis with 80% receiving no anticonvulsants and 1% receiving prophylactic anticonvulsant therapy for less than seven days, putting them at higher risk for early PTS; 11% of patients did receive seizure prophylaxis, but for more than seven days. As seizure prophylaxis has not been found to be effective in preventing late seizures occurring after seven days post-TBI, the continuation of seizure prophylaxis identified in this group of patients incurs higher risk for adverse effects and greater cost without providing any additional benefits in seizure prevention [8,14,15]. Taken together, the proportion of patients receiving the recommended seizure prophylaxis in this study is lower than that found by others. In survey studies, self-reported use of the recommended seven-day seizure prophylaxis ranges from 38 to 50% [14,23]. Even though 26-65% of TBI patients were found to have been started on seizure prophylaxis in studies that employed medical record reviews, the duration of anticonvulsant use was not reported in two of these three studies [24-26]. Thus, determining whether or not the prophylaxis was in line with recommendations is not possible, making it difficult to directly compare to the current study. The one study that did comment on the duration of anticonvulsant use found that almost one-quarter of patients started on seizure prophylaxis continued it for more than the recommended seven days, meaning that only one-third of patients received the recommended seizure prophylaxis at best [24]. The lower rate of recommended seizure prophylaxis in the current study confirms the high level of variability in practice and highlights the importance of objectively and quantitatively describing post-TBI anticonvulsant use to accurately understand local practice patterns and make targeted improvements to patient care. 

The proportion of patients receiving the recommended seven-day seizure prophylaxis did not increase after the release of the INESSS-ONF guidelines. This was surprising given the abundant supportive evidence and the simultaneous publishing of similar recommendations in the Brain Trauma Foundation’s guidelines for severe TBI [4]. The low uptake of the guideline recommendations found here may be influenced by a number of factors.

To investigate which patient factors might influence seizure prophylaxis practices, demographics and injury characteristics in patients receiving and not receiving the recommended seizure prophylaxis were compared. Age, gender, injury severity and mechanism of injury did not differ significantly between the two groups, consistent with previous studies [3]. In both groups, there were more severe than moderate TBIs, and the most common mechanism of injury was falls. Overall, this study’s findings suggest that these patient factors were not a driving force behind the decision to initiate seizure prophylaxis post-TBI.

Other factors may have influenced guideline uptake. A recent systematic review identified five main areas influencing guideline implementation: the guideline itself, the target healthcare professionals, patient characteristics, the work environment and the type of implementation strategy [27]. In the case of the INESSS-ONF recommendations for seizure prophylaxis, a lack of awareness among healthcare providers may be playing a role. Whereas the INESSS-ONF guidelines focus on rehabilitation, seizure prophylaxis is part of the acute management of TBI. As acute care was primarily overseen by neurosurgeons in this study, and not by rehabilitation specialists, there may have been a lack of awareness about the guidelines. This idea is also supported by the finding in this study that, though a preprinted order set existed for severe TBI and included appropriate seizure prophylaxis, it was never used. Uptake may be improved if the guidelines are disseminated more broadly or if rehabilitation specialists are involved earlier. Factors related to the guideline itself may also be playing a role. Primarily, seizure prophylaxis recommendations were not identified as priority recommendations, which may have decreased the uptake and level of awareness. Secondly, the recommendation is qualified with level C evidence, which, given the longstanding controversy surrounding seizure prophylaxis, may not have been convincing enough to change clinical practice. This is in line with a recent study of the implementation of stroke rehabilitation guidelines, which found agreement with the guideline recommendation to be a key factor influencing uptake [28].

The use of seizure prophylaxis may also increase with increasing evidence supporting its effectiveness in decreasing rates of early PTS. Hence, this study also recorded the occurrence of PTS. Rates of early PTS were low, consistent with previous studies [2-5]. There was no statistically significant difference in the proportion of patients who suffered early PTS when comparing the patients who did and did not receive the recommended seizure prophylaxis. However, given the low event rate, this lack of statistical significance should be interpreted with caution.

The analysis of secondary outcomes in this study is limited by sample size and low event rate. Though it was adequately powered to identify clinically significant changes in the uptake of recommended seizure prophylaxis, it may be underpowered to detect differences in the PTS rate due to the small number of patients in the seizure prophylaxis group and low incidence of PTS. Another potential limitation of this study is that it included only patients admitted to a single centre with TBI; hence, the practices we discussed may not be generalizable to all centres. However, given the wide practice variability, it is important to understand local practices before devising strategies to improve patient care. As this centre was a level-1 trauma centre, there is also a potential for bias toward more severe injuries. However, the majority of patients with a moderate to severe TBI are admitted to hospitals; hence, the study cohort should represent a significant proportion of the target study population, and one would not expect a systematic difference in injury severity between the compared time periods; we did not find any either. Finally, this study compared the 11 months immediately preceding and following the INESSS-ONF guideline release, which may have resulted in lower than expected implementation of the recommended seizure prophylaxis, factoring in an expected delay between guideline release and subsequent dissemination and uptake of the recommendations. However, the INESSS-ONF collected implementation data for a subset of recommendations, not including seizure prophylaxis, over a similar timeframe and found that the majority were being implemented, suggesting that it is reasonable to expect uptake of the recommendations in this study’s timeframe [29].

## Conclusions

Overall, this study found that the majority of patients with moderate and severe TBI at our level-1 trauma centre did not receive the recommended seven-day seizure prophylaxis post-injury and that the release of the INESSS-ONF guidelines did not change the practice. These findings highlight the wide practice variability in post-TBI seizure prophylaxis and the importance of understanding local practice patterns to inform quality improvement. The findings further indicate that strategies should be identified to encourage increased seizure prophylaxis implementation. A comparison of patients receiving and not receiving prophylaxis suggests that patient and injury factors do not influence the use of seizure prophylaxis. Further studies may focus on identifying other factors influencing post-TBI seizure prophylaxis practices, including provider and guideline factors, to improve patient care.
